# Myostatin and the Heart

**DOI:** 10.3390/biom13121777

**Published:** 2023-12-12

**Authors:** Małgorzata Knapp, Elżbieta Supruniuk, Jan Górski

**Affiliations:** 1 Department of Cardiology, Medical University of Białystok, 15-276 Białystok, Poland; 2 Department of Physiology, Medical University of Białystok, 15-222 Białystok, Poland; elzbieta.supruniuk@umb.edu.pl; 3 Department of Health Sciences, University of Łomża, 18-400 Łomża, Poland; gorski@umb.edu.pl

**Keywords:** myostatin, heart physiology, myocardial infarction, cardiac hypertrophy, chronic heart failure, cardiac cachexia

## Abstract

Myostatin (growth differentiation factor 8) is a member of the transforming growth factor-β superfamily. It is secreted mostly by skeletal muscles, although small amounts of myostatin are produced by the myocardium and the adipose tissue as well. Myostatin binds to activin IIB membrane receptors to activate the downstream intracellular canonical Smad2/Smad3 pathway, and additionally acts on non-Smad (non-canonical) pathways. Studies on transgenic animals have shown that overexpression of myostatin reduces the heart mass, whereas removal of myostatin has an opposite effect. In this review, we summarize the potential diagnostic and prognostic value of this protein in heart-related conditions. First, in myostatin-null mice the left ventricular internal diameters along with the diastolic and systolic volumes are larger than the respective values in wild-type mice. Myostatin is potentially secreted as part of a negative feedback loop that reduces the effects of the release of growth-promoting factors and energy reprogramming in response to hypertrophic stimuli. On the other hand, both human and animal data indicate that myostatin is involved in the development of the cardiac cachexia and heart fibrosis in the course of chronic heart failure. The understanding of the role of myostatin in such conditions might initiate a development of targeted therapies based on myostatin signaling inhibition.

## 1. Background

**Figure 1:**
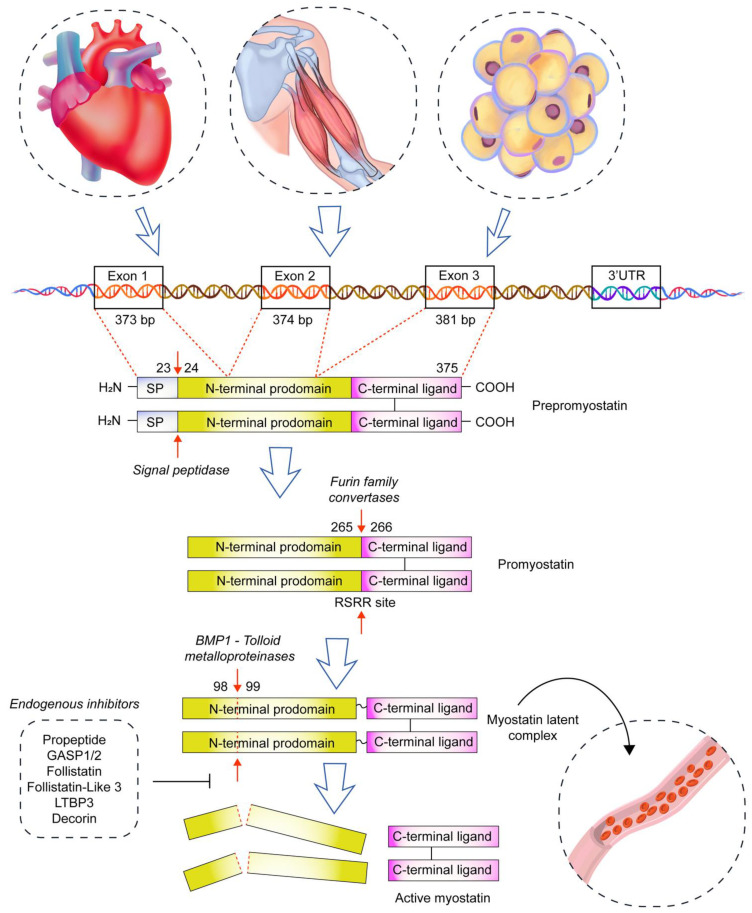
The pathway of free myostatin formation. The myostatin gene is located on chromosome 2 in humans and is composed of three exons and two introns. Prepromyostatin exists as a disulfide-linked homodimer composed of a signal peptide (SP), N-terminal prodomain, and C-terminal ligand. It is later processed by signal peptidase, furin protein convertase, and BMP-1/tolloid proteinases. In the blood, myostatin is present in the form of latent complex; thus, only one step is needed to release free myostatin. In skeletal muscles, promyostatin is secreted mostly in the interstitial fluid; thus, two steps are needed to release free myostatin. Blood myostatin is inhibited by follistatin, follistatin-related gene, propeptide, GASP 1 (growth and differentiation factor-associated serum protein 1), and LTBP3 (latent TGF-β binding protein 3). Blue arrows indicate the direction of myostatin synthesis, while red arrows show the place of enzyme action.

Skeletal muscle is now considered an endocrine organ based on its secretion of a number of bioactive factors collectively called myokines [1,2]. Profiling the composition of the muscle secretome has identified the presence of myostatin (growth differentiation factor-8, GDF-8), a member of the transforming growth factor β superfamily (TGF-β), among the secreted growth factors. In 1997, myostatin was discovered to be expressed in the skeletal muscles during both embryonic development and in adult mice [3]. In addition, small amounts of this protein are produced and released by the adipose tissue [3] and myocardium [4,5]. Myostatin is secreted as promyostatin, which is biologically inactive; thus, it becomes sequestrated in the extracellular matrix, where it undergoes two-step proteolytic cleavage. First, promyostatin is converted to latent complex by the enzyme furin protein convertase; this complex is inactive as well. It is then converted to a biologically active form by BMP-1/tolloid proteinases. In plasma, myostatin is present as a latent complex; and thus, it is activated in one step only by BMP-1 [6,7,8]. In addition to the propeptide that controls myostatin activity, circulating myostatin is regulated mostly by other inhibitory binding proteins, mostly propeptide, follistatin, and follistatin-related gene (Figure 1) [9,10,11,12]. 

Myostatin present in the interstitial fluid acts locally in the autocrine/paracrine way, while upon release into the blood stream reaches different remote tissues to exert systemic effects. The effects of myostatin depend on complex signal transduction mechanisms involving the activation of several downstream pathways and their precise integration. The C-terminal dimer of mature myostatin binds to activin type II B receptors (ActRIIB). This leads to the activation of the Smad 2 and 3 transcription factors (the canonical Smad pathway), which inhibits protein synthesis and activates protein degradation [6,7,8]. Myostatin additionally activates other intracellular pathways (noncanonical pathways), including, for instance, the suppression of Akt signaling cascade [7,8,10]. Myostatin can act on skeletal myocytes indirectly, namely through the satellite cells. These cells reside between the sarcolemma and basal lamina of a myocyte in a dormant state. When the myocyte is damaged (e.g., during eccentric exercise), the cells “wake up” and proliferate. They then fuse with the damaged myocytes and give them nuclei, leading to the enlargement of the myocytes (hypertrophy). The activated satellite cells can merge and form new myoblasts (hyperplasia). Part of these newly proliferated cells become dormant, replenishing the cells used for proliferation [13,14,15,16,17]. Myostatin inhibits proliferation of the satellite cells, thereby inhibiting muscle enlargement during strength training [7,8,18]. One report shows that myostatin may act prior to the activation of the satellite cells [19]. 

Myostatin’s function in skeletal muscle has become identified with inhibiting growth, while its expression in the heart indicates that it has additional non-atrophy stimulating functions. The evidence suggests that cardiac-derived myostatin is important for cardiogenesis, although controversies exist with respect to its role in the adult heart. During pathological loading of the heart, increased cardiac production of myostatin may be maladaptive, considering that it has systemic effects associated with cachexia. We have yet to fully determine myostatin disease-specific functions, which may have both systemic and local implications. The current knowledge on such interplay is discussed in this review, with points requiring further clarification, such as sex-specific alterations, being particularly stressed.

## 2. Myostatin and Skeletal Muscles

As indicated above, the serum myostatin level in healthy individuals depends mostly on its secretion by skeletal muscles. Therefore, it seems reasonable to outline the regulation of production and secretion of myostatin by skeletal muscles before discussing the role of the protein in the functioning of the heart.

Skeletal muscles are composed of different types of fibers classified based on the characteristics of energy metabolism and properties of myosin heavy chain. In rats, three types of fibers are distinguished: slow twitch-oxidative (type I, red), fast-twitch oxidative-glycolytic (type IIA, red), and fast-twitch glycolytic (type IIX, white) [20,21]. Each type of fiber shows the expression of myostatin. However, the mRNA expression in the white fibers is about three times higher than in the red fibers [5]. In line with this, studies on C2C12 cells showed that myostatin mRNA was present mainly in differentiated myotubes containing myosin heavy chain II isoform, and was not expressed in myoblasts. Cellular localization was almost totally restricted to the nucleus, with only small amounts detectable in the cytoplasm, which suggests that myostatin may play a role in transcriptional regulation [22]. 

Genetic evidence conclusively demonstrates a role for myostatin as a potent negative regulator of muscle mass. Knockout of myostatin gene or inhibition of its action by antibodies results in a spectacular visible enlargement of skeletal muscle (so-called ‘double muscling’). It has been shown in animals including mice, rats, and different farm animals [3,23]. A report from 2004 described a case of skeletal muscle enlargement in a human newborn child that resulted from a mutation in the myostatin gene [24]. On the contrary, overexpression of myostatin in skeletal muscles reduces skeletal muscle mass while decreasing fiber size and number of nuclei in transgenic male mice, though not female mice [25]. Similarly, injection with myostatin-producing cells reduces skeletal muscle mass in mice, leading to wasting syndrome in the long run [26].

Experimental evidence shows that in addition to myostatin affecting muscle fiber-type composition, it is involved in the control of muscle function. It has been shown in mice that removal of myostatin results in a reduction in the number of type I fibers and elevation in the number of type IIX fibers in the soleus and the extensor digitorum longus (EDL) muscle [27]. Denervation has been shown to increase myostatin mRNA and protein expression in the gastrocnemius, which can contribute to their atrophy [28]. Loss of myostatin impacts the contractile properties of muscle fibers, although the magnitude of this modification depends on the fiber type. Its removal reduced maximal tetanic force in soleus (red muscle) and EDL (white muscle), with the reduction in the EDL muscle being more pronounced than in the soleus. This is explained by the two times higher number of ActRIIB in EDL as compared to the soleus [29], indicating that white muscle can be more susceptible to myostatin expression modulation. 

Even one session of resistance exercise reduces the plasma myostatin level and its expression in human skeletal muscles [29,30,31]. Additionally, submaximal exercise reduces myostatin expression in the muscles; however, this decrease was two times weaker as compared to the resistance exercise protocol [32,33]. In rat models, an acute bout of swimming resulted in a marked, though insignificant, reduction in myostatin expression in the white fibers present in the gastrocnemius muscle while having no effect in the red fibers present in the same muscle [5]. Four-week swimming training significantly reduced the myostatin mRNA level in both fiber types present in the gastrocnemius [5]. Data obtained in humans showed reduced expression of myostatin mRNA in skeletal muscle and plasma after resistance strength training [34,35,36]. However, an elevation in the myostatin concentration in serum and muscle after 12 weeks of very heavy training has been reported as well. The difference was ascribed to the severity of training, although inhibitors of myostatin can be involved to overcome its suppressing effect on muscle hypertrophy [37]. Contrary to exercise, hypokinesia increased expression of myostatin in human muscle after 25 days of bed rest [38] or 28 days of ankle joint disuse [39]. It was additionally elevated in different rat skeletal muscles after unloading [40] and after 17 days of stay in the microgravity environment of the NeuroLab space shuttle flight [41].

Glucocorticoids and androgens exert opposite effects on skeletal muscle protein metabolism. The former activate proteolysis and reduce protein synthesis, thereby reducing the protein mass [42]. Androgens activate protein synthesis, increasing muscle mass [43]. Glucocorticoids increase myostatin mRNA expression in skeletal muscles, while knockout of the myostatin gene prevents muscles atrophy after dexamethasone treatment [44]. This would suggest that myostatin facilitates (at least partly) the proteolytic action of the hormone. Androgens stimulate myostatin production in the muscles [45], which may indicate that androgens inhibit excessive skeletal muscle enlargement stimulated by themselves. 

Another factor that influences myostatin levels in the body is obesity, along with other metabolic diseases. Both muscle and circulating myostatin expression have been shown to increase in obese human subjects, and higher protein release was noticed in myotubes derived from myoblasts obtained from muscle biopsies of obese compared to non-obese women [46]. This upregulation is reversed by gastric bypass surgery and resultant weight loss [47]. Furthermore, streptozotocin-induced diabetes elevated the mRNA expression of myostatin, follistatin, and ActIIB receptor in the white section of the gastrocnemius muscle [48].

It remains an open question why skeletal muscles secrete myostatin, the compound which inhibits their own enlargement. Bullough [49] formulated a theory saying that each organ produces a factor limiting its own growth, and called this putative factor a “chalone”. In support, Lee et al. [50] analysed the role of myostatin in skeletal muscles in detail and came to the conclusion that because myostatin acts as a negative feedback regulator of muscle mass, it can be regarded as a skeletal muscle chalone. 

The most recent completed phase 2 clinical trials (www.ClinicalTrials.gov accessed on 30 November 2023) have tested myostatin inhibitors such as RO7239361 (BMS-986089), PF-06252616 (domagrozumab), and SRK-015 to verify their potential to treat different neuromuscular conditions, including muscle dystrophy and spinal muscular atrophy. Nonetheless, these effects did not reproduce the promising results from animal models. Additionally, clinical studies have focused on the associations between myostatin and levels of molecules related to the paresis and progression of cancers and associated cachexia.

## 3. Myostatin and Heart Physiology

### 3.1. Myostatin and Heart Morphology

The results on a relationship between myostatin expression and heart weight are not uniform. Myostatin overexpression decreased the whole heart and left ventricular size in mice, whereas its removal had an opposite effect [51]. Interestingly, myostatin overexpression, specifically in skeletal muscles, decreased the heart mass only in male mice [25]. According to Rodgers et al. [52], hearts from adult male and female myostatin-null mice are larger than in wild-type mice. Both the heart and body weight of the neonates were heavier in the wild-type mice than in the myostatin-null mice, making the heart/body weight ratios similar in both groups. In myostatin-null mice, the left ventricular mass, internal diameters, and diastolic and systolic volume were larger than the respective values in the wild-type mice. These data would seem to indicate that lack of myostatin produces eccentric cardiac hypertrophy. In opposition to the above results, Cohn et al. [53] did not find any differences in body and heart weight or heart/body ratio between myostatin-deprived and wild-type mice. Thus, the question arises whether distinct compensatory mechanisms can account for the variations in results seen across studies. Gaining a deeper comprehension of myostatin biology is essential in order to characterize its physiological importance in the heart.

### 3.2. Regulation of Heart Myostatin Expression

The available data clearly indicate that myostatin is involved in the regulation of cardiomyocyte growth, proliferation, and functioning (Table 1). The presence of myostatin mRNA and protein have been shown for the first time in cardiomyocytes and Purkinje fibers of foetal and adult sheep, being significantly higher in the former [4]. Similar observations were noticed in rat myocardium, wherein myostatin expression depended on the age of development. The mRNA and protein myostatin expression in foetal cardiomyocytes was low, subsequently increased markedly up to the tenth day of life, and then decreased in the adults to the control level. More specifically, myostatin mRNA expression in the adults was fifteen-fold lower and myostatin protein expression was two-fold lower as compared to the expression on the tenth day [54]. Additionally, differences in myostatin expression between the heart ventricles were reported in growing piglets, which may reflect chamber-specific alterations in myocardium in response to workload. The expression in the left ventricle of 20-day-old piglets was three times higher than in newborn ones, whereas the expression in the right ventricle remained stable. The myostatin expression in the left ventricle of the newborn piglets was two times higher, and in the 20-day-old piglets was six times higher than in the right ventricle [55]. These latter data are in opposition to the data obtained in sheep [4]. It is difficult to say whether the difference in the direction of changes between sheep and the piglets should be ascribed to differences in the species or the time of sampling (i.e., adult sheep in [4] and 20 day old piglets in [55]). It seems likely that the growing level of myostatin in the left ventricle inhibits excessive activity of the genes responsible for the ventricle enlargement. To address the role of myostatin at early levels of cardiac development, exogenous myostatin was administered to cultured embryonic and early neonate cardiomyocytes. The inhibition of proliferation occurred in both cell lines, mainly via blockade in the G1-S phase of the cell cycle. The same study showed that myostatin inhibits protein synthesis in isolated cardiomyocytes [54]. Several underlying pathways through which myostatin interferes with cardiac growth have already been described. These involve the inhibition of phenylephrine (α1 adrenergic receptor activator)-dependent protein synthesis and heart growth as well as phenylephrine-induced growth of cultured cardiomyocytes [56] through the depression of p38 and the serine-threonine kinase Akt activity [57]. In accordance with the above, in myostatin knockout mice this phenylephrine-induced heart growth was potentiated as compared to the wild-type mice [56]. It was further proved that phenylephrine stimulates production of myostatin by cardiomyocytes [56]. Exogenous myostatin inhibited the action of IGF-1 and its analogue LR3 on proliferation of the cultured cells [52]. Altogether, these results clearly indicate that myostatin is responsible for inhibition of the hyperplastic growth, cardiomyocyte proliferation, and reduction in the rate of protein synthesis in growing heart.

Cyclic stretch. Cyclic stretching resulted in several-fold elevation in mRNA and protein expression of myostatin in neonatal rat cultured cardiomyocytes, with a concurrent increase in IGF-1 secretion. In the next step, the stretch-induced secretion of myostatin was prevented by the inhibition of IGF-1. Therefore, it was concluded that IGF-1 was responsible for induction of myostatin expression in the cardiomyocytes by cyclic stress [58].

Exercise. Eight weeks of either resistance or moderate intensity aerobic training did not affect the heart myostatin mRNA expression in rats. However, it markedly elevated heart mRNA follistatin expression. As a result, the ratio of follistatin mRNA/myostatin mRNA increased considerably [59]. Because follistatin is an inhibitor of myostatin, such a shift in follistatin/myostatin balance would facilitate the heart hypertrophy in the course of training. On the other hand, four weeks of swimming training was shown to increase mRNA myostatin expression in the heart by 74% [5]. Such an elevation in the myocardial level of myostatin could exert a direct inhibitory effect on development of heart hypertrophy during training. However, it is difficult to explain the reasons for the difference between the two reports. 

Angiotensin II. Angiotensin II exerts powerful effects on the cardiovascular system, for instance by promoting the development of cardiac hypertrophy. Initially, it serves as a compensatory mechanism to maintain hemodynamic homeostasis, although long-term angiotensin II upregulation becomes maladaptive and results in pathological cardiac hypertrophy [72]. It has been shown that angiotensin II promotes expression of myostatin in isolated rat neonatal cardiomyocytes. The process was mediated by the activation of the p38 MAP kinase and MF-2 pathways. This would suggest that myostatin is a negative feedback regulator of angiotensin II-induced heart hypertrophy [60].

Insulin-like growth factor 1 (IGF-1). IGF-1 increases the expression of myostatin in cultured cardiomyocytes [58]. Gaussin and Depre [73] suggested that because IGF-1 stimulates both cardiac growth and cardiac myostatin production, myostatin is a cardiac chalone of IGF-1. 

miRNA-208a. miRNA-208a is present in the heart and is involved in the pathogenesis of multiple cardiovascular diseases. Its overexpression in mouse heart reduces the expression of myocardial myostatin protein. Deletion of miRNA-208a leads to the elevation of myostatin expression [61]. This would seem to indicate that myostatin is likely to be involved in the action of miRNA-208a. On the other hand, removal of myostatin increases the expression of miRNA-208a both in vivo and in vitro. Myostatin treatment reduces the expression of miR-128 induced by the aorta coarctation and by angiotensin II in isolated cardiomyocytes [74].

### 3.3. Myostatin and Heart Function

The available data provide convincing proof that myostatin is needed for normal heart function. In experiments by Rodgers et al. [52], stroke volume, fractional shortening, and ejection fraction were all lower in myostatin-null mice than in wild-type animals. Reduction in end systolic volume, elevation in fractional shortening, and ejection fraction after treatment with isoproterenol (β-adrenergic agonist) were much greater in the myostatin-null mice than in their wild-type counterparts. These differences would be due to increased calcium release from sarcoplasmic reticulum in the myostatin-null mice. This establishes a mechanism for a myostatin-related maintenance in cardiac output, which otherwise could be expected to decline with high heart rate. On the contrary, Butcher et al. [75] did not find a difference in terms of the response of fractional shortening and ejection fraction after stimulation with isoproterenol or blockade of β-adrenergic receptors (with propranolol) between control and myostatin knockout mice. 

Selective deletion of myostatin in cardiomyocytes of adult mice increases lethality, and leads to heart failure and ventricular hypertrophy. However, in this model the skeletal muscle mass, myocyte area, and myostatin expression remained intact [76]. Long-term overexpression of myostatin in mouse heart reduces the ejection fraction and stroke volume, elevates the end systolic volume and end diastolic volume, and induces development of fibrosis [77]. Further studies of this group revealed that myostatin activates the TAK1-MKK3/p38 signaling pathway. Activation of this pathway increases production of collagen 1. A very important observation of the two studies was that within 6 weeks after the removal of myostatin from cardiomyocytes, its level in the heart and serum returned to normal. It was further shown that this adaptive mechanism resulted from the increased production of myostatin by non-cardiomyocyte cells present in the heart [76,77]. Other models of adaptation to prevent undesirable consequences of myostatin loss can be noticed in germ-line mutants unable to synthesize myostatin via non-cardiomyocytes. One possible mechanism could be a rise in GDF11 due to functional overlap between the two proteins (myostatin and GDF11) [78], meaning that only limited effects on cardiac function were observed. There are data indicating that removal of myostatin does not result in heart hypertrophy or affect cardiomyocyte size. Furthermore, it does not attenuate cardiac fibrosis in the dystrophin-deficient mdx mouse model of Duchenne muscular dystrophy. The major conclusion of the latter study was that myostatin does not function as a crucial regulator of myocardial growth and regeneration in cardiac muscle in vivo. It was claimed that the difference in the results with other reports was due to the use of better equipment to evaluate heart function, specifically, a high-resolution echocardiography apparatus [53].

### 3.4. Myostatin and Heart Metabolism 

The foetal heart mostly uses carbohydrates as an energy fuel, whereas adult hearts preferably use free fatty acids. Hypertrophied adult hearts are overly reliant on glucose as an energy source. This manifests in acceleration of glycolysis, increased uptake of glucose, and increased production of lactate [79]. APMK (AMP-activated protein kinase) is the key regulator of energy homeostasis in the heart [80]. Myostatin has been shown to modulate cardiac energy substrate reliance and limit the risk of heart failure. Inactivation of myostatin in adult mouse cardiomyocytes resulted in a nearly two-fold elevation of phosphorylation and activation of AMPK. As a consequence, an elevation in glucose uptake and glycolysis in the myocytes was noted. On the contrary, myostatin overexpression resulted in strong inhibition of AMPK phosphorylation and ultimately prevented a metabolic switch in the direction of glycolysis and glycogen accumulation [76]. Based on the above, a potential cardioprotective role of myostatin can be ascribed to the suppression of metabolic reprograming towards the fetal metabolic pattern that occurs during cardiac hypertrophy and maintenance of a mature aerobic energy metabolism.

Myostatin can counteract the pathological hypertrophic effects through the modulation of protein turnover in the myocardium. Exogenous myostatin increases proteolysis and reduces protein synthesis in cultured cardiomyocytes. A closer look at the mechanism of myostatin-activated proteolysis revealed that it phosphorylates Smad2 and Smad3 proteins, leading to the activation of proteolysis and the process of autophagy. Decreased protein synthesis was mediated by inhibition of Akt, Foxo3a, and P70S6K phosphorylation [81]. These data clearly indicate that myostatin is involved in regulation of the myocardial metabolism.

### 3.5. Myostatin and Heart Pathology

There are data available from animal and human studies indicating changes in the myocardial metabolism of myostatin in heart pathology as well effects of these changes on distant tissues, mainly skeletal muscle. The most investigated heart disorders are myocardial infarction, myocardial hypertrophy, and chronic heart failure. 

### 3.6. Myocardial Infarction

Experiments on animals. Coronary circulation is very poorly equipped in anastomoses; thus, collateral circulation does not play a protective role against cardiac ischemia. As a result, occlusion of a coronary artery results in acute ischemia and necrosis of the area fed by the occluded artery. In light of the data on the role of myostatin in heart physiology, it could be expected that myostatin might affect different parameters of the heart function after myocardial infarction. Indeed, the data obtained thus far both in animal experiments and from human beings confirms this assumption. In the reports cited below, the experimental myocardial infarction was produced by ligation of the left anterior descending coronary artery (LADA). Shrama was the first to show that the expression of myostatin protein in sheep was upregulated in the myocardium around the infarcted area [4]. In rats, 8 weeks after LADA ligation [62] myocardial myostatin mRNA expression increased only insignificantly; however, the expression of myostatin protein increased more than four times compared to the respective control values. Interestingly, four weeks of training prevented this elevation in the protein expression. Thus, the suppression of myostatin can be one of the factors of anti-catabolic effects of exercise training in chronic heart failure. Another protocol was employed by Rodgers et al. [52]. They studied myostatin expression in different parts of left ventricle 4 weeks after ischemia/reperfusion procedure. The ischemia lasted for 30 min, and afterwards the samples were harvested from infarcted, border-infarcted, and healthy regions of the myocardium. The expression of myostatin in different locations was similar, and was not affected by ischemia. However, the expression of follistatin was elevated only in the infarcted area. Such a shift in the myostatin/follistatin balance might indicate a reduction in the role of myostatin during recovery from the post-ischemia/reperfusion injury. Furthermore, a role of myostatin in post-infarction recovery was studied in myostatin-null mice 1 and 28 days after the infarction [82]. The infarct size in myostatin-null mice was similar to the infarct size in wild-type mice. However, deletion of myostatin reduced the post-infarct mortality rate by 20%. The heart rate in the myostatin-null group 28 days after the infarct was higher than in the wild-type mice. Among the parameters characterizing left ventricular function, fractional shortening and ejection fraction were reduced to a similar degree in both groups in one day after the infarct. However, 28 days after the infarction, both parameters normalized in the myostatin-deprived group only. Moreover, lack of myostatin partially prevented deposition of collagen and scarring, and preserved a greater viable area of the myocardium than in the wild-type mice. These results indicate that lack of myostatin is beneficial, as it partially reduced the consequences of myocardial infarction. 

A large amount of data on the level of myostatin and other compounds functionally related to myostatin in rat hearts and serum after myocardial infarction were presented by Castillero et al. [63]. These are the only data examining expression of these factors at several time points beginning from 10 min up to 2 months after myocardial infarction. As these data are unique, more details are presented here. It was found that the myostatin level in the heart increased as early as 10 min after the infarction, and partially normalized over the next hours. It markedly increased again 24 h after the infarction and remained elevated thereafter. The heart level of follistatin remained stable. Cardiac IGF-1 was elevated in the examined period of time, though this elevation was significant only one month after the infarct. Cardiac pAkt/Akt ratio increased after 24 h, remained elevated for 1 month, and then returned to the level in the sham-operated mice. Collectively, the upregulation of cardiac myostatin expression would counteract the pro-hypertrophic action of IGF-1 and pAkt/Akt ratio after infarction. Moreover, MAP kinase and P-p38/p38 ratio were elevated at 10 min and one hour, and then from one week onwards. The cardiac pSmad2,3/Smad2,3 ratio fluctuated, being significantly elevated at 2, 6, and 12 h as well as at one month after the infarct, indicative of the activation of muscle atrophy. Therefore, this study showed that infarction changes the balance between pro- and anti-hypertrophic factors in the myocardium. Interestingly, these alterations are not confined to the heart, as an elevation in the serum myostatin level was observed as well. Most likely, this was a consequence of activation of its production in the myocardium. Serum levels of myostatin increased already only ten minutes after the infarct, and remained elevated until the end of observation. The serum follistatin level was elevated from 24 h onwards, while the circulating IGF-1 concentration was not affected by the infarction. These data clearly indicate that changes in the levels of the examined parameters, and as such their effects on the heart’s post-infarct recovery, appear very early and are long-lasting. 

Human data. Data on the myostatin serum concentration after myocardial infarction are available in humans as well. In a study by Oliveira et al. [64], 102 patients with STEMI were included. STEMI was defined as ischemic chest pain with elevation of the ST segment of the electrocardiogram. Myocardial infarction reduced serum myostatin concentration as compared to the healthy control group. The mortality rate among patients with lower serum myostatin concentration was higher than among those with less reduced levels. No association between serum myostatin and creatine phosphokinase (CK-MB) peak concentration or ejection fraction was shown. The reason for the difference in the direction of the change in the post-infarct serum myostatin concentration between rats (increase) and humans (reduction) remains an open question. 

The serum troponin I peak is regarded as a reliable indicator of infarction size. Meloux et al. [83] examined an association between serum myostatin and troponin I peak concentration in 296 patients with acute myocardial infarction. The serum myostatin data were positively correlated with troponin I peak data. We are not aware of any other results comparing the serum myostatin level and troponin I peak as markers of the infarct size. The data presented by this study are encouraging. Undoubtedly, further studies are needed to confirm the usefulness of serum myostatin levels in evaluating infarct size. In the same study [83], the serum myostatin concentrations were higher in patients with ventricular tachycardia or fibrillation acquired in the hospital. This would seem to indicate involvement of myostatin in cardiac pathologies. Obviously, more data are needed on the relationship between myostatin and myocardial infarction in humans.

### 3.7. Myocardial Hypertrophy

Usually, two models of heart hypertrophy are used in experimental animals. In one model, the abdominal aorta is transversely constricted. This forces the left ventricle muscle to exert more work to pump blood through the narrowed vessel. As a result, left ventricle hypertrophy develops. In another model, an aortal–caval shunt is made. It increases the blood volume moving to the heart, which finally reaches the left ventricle. In turn, more work on the part of the left ventricle is required to pump the blood out to the aorta. As such conditions last for longer periods of time, the left ventricle muscle mass increases gradually, i.e., hypertrophy develops. Persistent increased workload finally leads to chronic cardiac failure. Shyu at al. [66] produced cardiac hypertrophy in rats using the volume-overload model for 4 weeks. The shunt increased heart weight and heart rate, left ventricular end-diastolic pressure, left ventricular end-diastolic dimension, and end-systolic dimension. At the same time, myostatin protein and mRNA expression in the myocardium increased more than twofold. Treatment with carvedilol (a nonselective blocker of β-adrenergic receptors and α1-adrenergic receptors) blocked the elevation of myostatin mRNA expression in the heart and skeletal muscle produced by the shunt. These latter data suggest that the adrenergic system is involved in the activation of myostatin expression in the muscles. No further data on this topic are available. 

Hypertension. Hypertension is another cause of the heart hypertrophy. Hypertension requires more work by the left ventricular muscle to pump blood out to the aorta against the increased blood pressure in the vessel, leading to hypertrophy of the muscle. Spontaneously hypertensive rats with heart failure showed a hypertrophied and dilated left atrium and left ventricle chamber, increased myocyte diameter, and increased myocardial interstitial collagen content. Left ventricular dysfunction manifested with decreased midwall fractional shortening. Concomitantly, a reduction in myocardial protein myostatin and follistatin expression in the left ventricle was demonstrated. The expression of both myostatin and follistatin was correlated with the functional parameters of the left ventricle [65]. These results are at odds with data reporting elevated myostatin expression in hypertrophied hearts [66]. The only cause for the difference between the two reports would different pathomechanisms of spontaneous hypertension compared to the experimental hypertrophy. It is likely that other factors accompanying spontaneous hypertension/hypertrophy could reverse the direction of the myostatin expression.

As indicated above, experimentally hypertrophied myocardium produces more myostatin. This raises the question of how prolonged exposure to elevated endogenous myostatin levels influences the myocardium itself as well as the skeletal muscles. An extensive study on this topic was performed by Qi et al. [74]. Cardiac hypertrophy was produced in rats by a reduction in the abdominal aorta diameter. The function of the heart was evaluated ten weeks later. Myostatin deletion augmented hypertrophy and autophagy produced by aorta coarctation or angiotensin II. This was accompanied by a reduction in the parameters of the heart function. Mechanistic studies on isolated cardiomyocytes revealed that myostatin silencing augmented the hypertrophic effect of angiotensin II. On the contrary, treatment with myostatin dramatically reduced both hypertrophy and autophagy of the cells, partially via inactivation of the AMP-activated kinase (AMPK)/mammalian target of rapamycin (mTOR) pathway and activation of the peroxisome proliferator activated receptor gamma (PPARγ)/nuclear factor κB (NF-κB) pathway. Myostatin treatment reduced expression of miR-128 induced by aorta coarctation and angiotensin II in isolated cardiomyocytes. The final conclusion was that increased production of myostatin is a self-defence mechanism in the hypertrophic heart that leads to the attenuation of hypertrophy, heart dysfunction, and autophagy. 

### 3.8. Chronic Heart Failure in Humans

Chronic heart failure (CHF) is an incurable disease and leads eventually to death. Chronic heart failure may lead to cachexia. The most frequent causes of chronic heart failure are myocardial infarction, long-term hypertension, cardiomyopathy, and valvular heart disease. 

**Figure 2:**
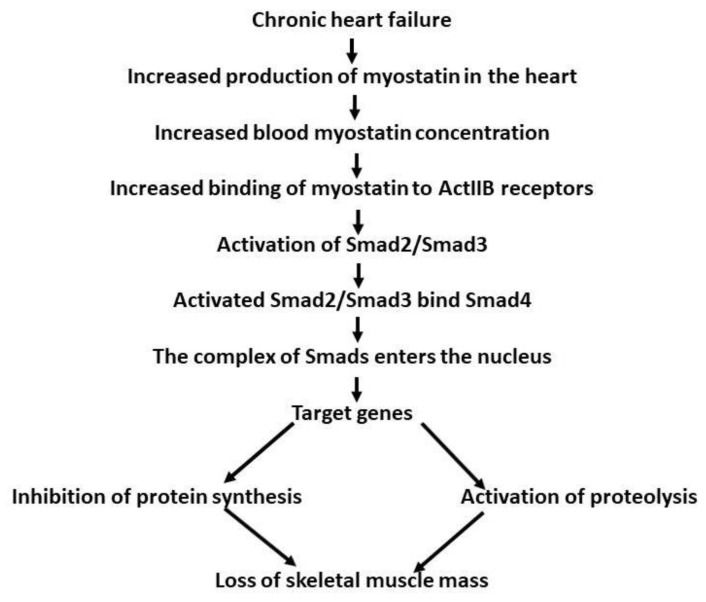
The sequence of events in chronic heart failure leading to development of cardiac cachexia on the Smad2/Smad3 (canonical) pathway. Myostatin inhibits Akt, leading to dephosphorylation of FOXO. Dephosphorylated FOXO enters the skeletal muscle nucleus and increases transcription of atrophy genes. Inhibition of Akt downregulates the mTOR pathway of protein synthesis [6,8].

There are contrasting data on serum myostatin level in patients with CHF. Furihata [67] examined 31 men with CHF and showed that myostatin serum levels were reduced, whereas the level of follistatin was elevated compared to the control group. The reduction in serum myostatin concentration was associated with muscle wasting in the lower extremities. Chen et al. [68] examined 288 patients with CHF treated in hospital. They observed a gradual elevation in serum myostatin concentration with advancing gravity of the disease, being highest in the class IV patients (according to criteria of the New York Heart Association-NYHA). During 51 months of follow-up, elevated mortality and hospitalization rates were recorded among those patients with high serum levels of myostatin. This was interpreted as being due to serum myostatin concentration being an independent predictive factor of survival in patients with CHF. The difference between the two reports was ascribed to differences in the condition of the patients in the two studies. Furihata et al. [67] examined CHF patients with compensated heart failure, 70% of whom were subjected to exercise training, whereas the patients studied by Chen et al. [68] were admitted to the hospital in a decompensated state. Gruson et al. [69] reported elevated serum myostatin levels in 76 patients with stable congestive heart failure. No significant differences were observed between patients with CHF of ischemic origin and those with dilated cardiomyopathy. Elevated serum myostatin levels in patients with heart failure was reported by Chiang et al. [70]. The myostatin serum level was correlated with myocardial scarring. It was suggested that the plasma myostatin concentration could be a predictor of the level of scarring of the myocardium. Elevation myostatin expression in the blood in both animal and human heart pathology puts forward the question of its source, with the available data indicating that it comes from the heart itself. If this is the case, however, then why does pathologically changed myocardium produce more myostatin? One likely reason could be similar to that observed in cyclic stretching [58], that is, the heart changes its dimensions cyclically during the contraction/relaxation cycle. Changed heart muscle mass in hypertrophy or chronic heart failure creates new conditions for mechanical work, and could result in increased production of myostatin. Moreover, increased heart mass could secrete more myostatin. Increased production of myostatin would be expected to result in elevation of the concentration of the compound in the myocardial extracellular space. Thus, more myostatin would be available to bind to the ActRIIB present on the cardiomyocyte plasma membrane. This would increase the inhibitory action of myostatin, preventing/slowing down the myocardial enlargement. Therefore, the increased secretion of myostatin in heart pathologies would be a self-defence mechanism protecting against excessive cardiac enlargement. Obviously, accumulation of myostatin in the heart intracellular space would result in increased release of the compound in the blood and subsequent elevation in the blood myostatin concentration, which would in turn contribute to skeletal muscle wasting (Figure 2).

There is only a small amount of data on myostatin behaviour in the myocardium of patients with CHF. To clarify the problem, and because of the uniqueness of the samples, additional clinical data and results are included in our discussion of this issue. The human heart samples were harvested during heart transplantation. In a study by Fernández-Solà et al. [84], heart samples were collected from 66 heart donors with cardiomyopathy. The control group included people who had died suddenly, who were otherwise healthy, and whose hearts were not suitable for transplantation. Cardiomyocyte and nuclei size in patients with hypertrophy were enlarged. The expression of myostatin in the myocardium in hypertensive donors with cardiomyopathy was significantly higher than in non-hypertensive healthy donors. Further data [71] showed differences in myostatin levels between female and male patients with advanced heart failure. The myocardial samples were taken from the hearts after their removal to make room for the transplanted donor’s heart. Heart samples from healthy donors were taken after brain damage and confirmation that they were unsuitable for transplantation. It was found that the expression of mature forms of myostatin (25 kDa) and pSmad2 protein in females with heart failure were elevated by 1.9- and 2.5-fold, respectively, as compared to healthy female donors, and were higher compared to the respective data in male patients. In males with heart failure, only the level of pSmad2 was elevated (2.8-fold). The latent form of myostatin (50 kD) was reduced with heart failure in both sexes. Finally, the expression of ActRIIB was not affected by heart failure in either sex. Based on the above, the elevated expression of myostatin in females with chronic heart failure could predispose them to earlier development of cardiac cachexia. The difference in myostatin expression between female and male patients requires comment. It was previously found in mice that removal of the myostatin gene resulted in increase in the body mass by about 30% in both sexes [3]. As indicated above [25], overexpression of myostatin specifically in skeletal muscles reduces the examined skeletal muscle mass and heart mass only in male mice, not females. Hearts from adult male and female myostatin-null mice are larger than in wild-type mice; however, no difference in gain of heart mass between sexes was seen [52]. In the discussed study [71], myostatin expression in the myocardium of patients with heavy chronic heart failure was elevated only in females, not males. This is the first report on the heart myostatin expression in different sexes. As the female patients were at the postmenopausal age, a role of female hormones can be excluded. Certainly, it should be expected that the blood concentration of androgens in the female patients be lower than in male ones. One available study showed increased myostatin content in skeletal muscles after treatment with androgens [45]. Therefore, an elevation in the myocardial content of myostatin it could be expected in males rather than females. However, the results indicate the opposite. No reliable explanation of these phenomena can be offered at presence.

Thus far, only one report has been published on myostatin (and IGF-1) signaling at the end stage of heart failure (class III-IV according to NYHA classification) in humans with ischemic or dilated cardiomyopathy [85]. The control group consisted of healthy donors whose hearts had unsuitable clinical status. The heart samples were taken from the left and right ventricle and the septum from two groups of patients: organ donor patients and patients’ explanted hearts. The expression of myostatin mRNA, myostatin ActRIIB, IGF-1 and its receptor, and miR-208 were measured. In the healthy control group, there were no significant differences between the expression of myostatin, ActRIIB, and myostatin signaling index (myostatin x ActRIIB) between the examined samples from different heart parts. However, the control group combined three female and two male participants, which may have impacted the findings. Nevertheless, expression of IGF-1 mRNA and its receptor in the right ventricle were insignificantly higher than in the other parts of the myocardium. Calculation of the ratio of myostatin to IGF-1 and the ratio of their respective receptors’ expressions showed higher values for the left ventricle than for the right. Thus, myostatin overexpression may compensate for increased IGF-1-induced cardiac growth to mitigate excessive hypertrophy. Surprisingly, the effect of heart failure on the studied parameters depended on the aetiology of the failure. In dilated cardiomyopathy [85], the myostatin mRNA expression increased about two-fold in each part of the myocardium, whereas the expression of ActRIIB remained unchanged. IGF-1 expression was elevated only in the right ventricle. In patients with ischemic heart failure, the level of these parameters did not differ from the appropriate values in the control group. It is likely that the reduced supply of oxygen in ischemic cardiomyopathy prevents the activation of myostatin expression seen in dilated cardiomyopathy. The meaning of these intra-cardiac differences is not clear. It is possible that the high expression of myostatin mRNA in the left ventricle leads to an elevation of the myostatin protein level, which in turn would lead to a reduction in the rate of growth of the muscle. At the present state of knowledge, it is difficult to find a reliable explanation of the difference between the ventricles. We assume that if myostatin activation in ICM patients occurs at different levels in both ventricles (mRNA or protein), then this response would be missed by only measuring gene expression. The regional asymmetry could be associated with the differences in pressure load in both ventricles. Subsequently, molecular make-up might predispose patients to either concentric or eccentric remodeling in the left and right chambers, respectively. It may be worth mentioning that our previous studies in adult rats have shown differences in the level of the lipolytic complex between the left and right ventricles in response to exercise [86] and tachycardia [87]. 

In another study, human left ventricle samples were collected before and after left ventricular assist device (LVAD) support in patients with advanced ischaemic and non-ischaemic dilated cardiomyopathy heart failure (ICM and DCM, respectively) [88]. Control samples were obtained from hearts disqualified for transplantation. Blood was collected from healthy people and from patients with stable DCM. Several differences were observed between the two groups of patients regarding both pre- and post-LVAD support. In the serum, myostatin latent complex expression in patients with DCM was over two times higher than in the healthy group. The content of circulating myostatin propeptide (a marker of myostatin activation) was low, however, and no difference between the groups was seen. However, these results in the blood did not mirror those for myocardial tissue. In the heart, the expression of myostatin propeptide was elevated in both the ICM and DCM groups before and after LVAD as compared to the control. This was probably triggered by myocyte stretching and higher myocardial stress. Because there were no concomitant changes in full-length myostatin content, this suggests a mechanism of self-renewal of myostatin to compensate for the cleavage of its portion in the muscle. Furthermore, myostatin activation in all the groups was supported by the rise in BMP-I expression and pSmad/Smad ratio. Among the DCM groups, myostatin propeptide and pErk/Erk ratio were higher in the post-LVAD group as compared to the pre-LVAD group. Importantly, the levels of myostatin propeptide in DCM patients was correlated with the changes in myocyte size. It was observed that myocyte diameters in patients with DCM were higher than in the control group, while the cell size was reduced after LVAD. Therefore, increased myostatin activity may contribute to this reduction in cardiomyocyte size in the DCM group after unloading. Such changes were not seen in ICM patients. While LVAD removed the pressure–volume overloading stimulus in both the studied groups, large scarring in ICM can lead to a lower rate of myocardial recovery after LVAD support. 

### 3.9. Cardiac Cachexia

According to Fearon et al. [89] cachexia is “a multifactorial syndrome defined by an ongoing loss of skeletal muscle mass (with or without of fat loss) that cannot be fully reversed by conventional nutritional support and leads to progressive functional impairment”. Heart cachexia develops in about 15% patients suffering from chronic heart failure in the terminal stage of the disease [90]. The mechanisms leading to the development of heart cachexia are not fully recognized [91,92]. Discovery of a role of myostatin in reduction in skeletal muscle mass called attention to its role in the development of cachexia. There are data indicating that increased production of myostatin in the myocardium may be at least partially responsible for cachexia developing during heart failure. In a study by Lima et al. [93], expression of myostatin and follistatin mRNA and protein in rat soleus and gastrocnemius were measured six months after myocardial infarction, when the rats developed chronic heart failure. The cross-sectional fiber area and follistatin were reduced only in the soleus. Myostatin. expression was not reduced. These results show differences in the susceptibility of particular muscle types in response to chronic heart failure. Castillero et al. [63] observed higher expression of atrophy markers in the gastrocnemius after myocardial infarction. The level of myostatin in the muscle was stable, while the level of plasma myostatin was elevated. Their conclusion was that the plasma, instead of local myostatin, triggered activation of the muscle atrophy. This conclusion is in line with reasoning by Breibart et al. [94]. Very strong support for a role of myostatin in the development of heart cachexia in rats and mice was provided by Heineke et al. [95] through the use of two models with either skeletal muscle-restricted or heart-restricted myostatin deprivation Deletion of myostatin from skeletal muscles resulted only in pronounced hypertrophy of the muscles. This indicates that the myostatin present in the muscles inhibited their growth. Removal of myostatin specifically from the heart did not affect the size of either the heart or skeletal muscles. This in turn suggests that under normal conditions extra-muscle myostatin does not control either skeletal muscle or heart mass. In the next step, aortic constriction pressure overload was introduced in mice and heart failure was diagnosed by echocardiogram. Afterwards, plasma myostatin expression increased 2–3-fold in the wild-type mice but not in the myostatin-null mice. This indicates that the increased plasma myostatin in the pressure-overloaded mice originated from the heart. Myostatin expression in the gastrocnemius muscle was not affected. The skeletal muscle mass (quadriceps, gastrocnemius, and soleus) was reduced in the heart failure group as compared to the control group. However, the muscle mass remained unchanged in mice whose hearts were deprived of myostatin. In another group of mice, myostatin was overexpressed in cardiomyocytes, resulting in a 3–4-fold elevation in plasma myostatin expression and reduced skeletal muscle and heart mass. Finally, the control and high-pressure groups were treated with myostatin antibodies (JA-16) beginning 8 weeks after the surgery; 6 weeks of treatment with antibodies did not affect either the mortality rate or heart performance while preventing skeletal muscle weight loss. The results presented above unanimously support the hypothesis that increased production of myostatin in the failing heart is at least partially responsible for the development of heart cachexia.

The results obtained in animal-based experiments clearly indicate a likely role of myostatin in the development of cardiac cachexia. However, these experiments were all performed on otherwise healthy animals. The results in human patients may be affected by a number of other factors involved in the development of cardiac cachexia that could be activated during diseases and treatments accompanying chronic heart failure. Moreover, for obvious reasons chronic heart failure is much shorter in rats than in humans. Therefore, the animal data must be interpreted with great caution in relation to human data. The human data are mostly descriptive. Undoubtedly, they have broadened our knowledge on the mechanism of cardiac cachexia, and thereby facilitated the development of specifics which might be useful in treatment of this disease. It is known that pre-clinical and clinical tests employing anti-myostatin antibodies and other anti-myostatin compounds are currently underway [6,8,96].

## 4. Conclusions

The temporal- and regional-specific control of myostatin and the expression of its associated proteins in both the healthy and infarcted heart contribute to the discrepant outcomes on its role in the heart. Despite this, most data show that removal of myostatin results in myocardial hypertrophy, whereas its overexpression reduces the heart mass. The expression of myostatin in the heart increases with progression of hypertrophy, and may inhibit its excessive growth. The available results clearly indicate that myostatin is responsible for the inhibition of hyperplastic growth, cardiomyocyte proliferation, and reduction in the rate of protein synthesis in the growing heart. Expression/activity of myostatin in the heart/cardiomyocytes is regulated by factors such as follistatin, angiotensin II, IGF-1, deletion of miRNA-208a, and cyclic stretching. This suggests the high flexibility of cardiac tissue with regard to monitoring and adjusting myostatin activity, although it can be disrupted by heart failure. Removal of myostatin results in eccentric cardiac growth and increases the susceptibility of the heart to stimulation by β-adrenergic receptors, which resembles the physiological hypertrophy found in endurance-trained athletes. These outcomes would be clinically beneficial in patients with severe ventricular dysfunction. On the other hand, heart pathologies such as infarction, hypertrophy, and chronic heart failure increase myocardial expression of myostatin. Genetic elimination of myostatin-mitigated cardiomyocyte death and myocardial interstitial fibrosis as well as preserved left ventricle function have been shown in mice. There is good evidence showing that increased prolonged production of myostatin by the myocardium in the context of chronic heart failure may ultimately lead to the development of cardiac cachexia. Many attempts, including pre-clinical and clinical trials, have been made to develop anti-myostatin procedures in treatment of skeletal muscle wasting in different diseases; however, as was recently stated by Nielsen et al. [97], these have met with “limited success”. Evidently, new approaches have to be used to continue the efforts in this very promising area. It appears that it is necessary to compare the efficacy of different myostatin-focused strategies (e.g., myostatin-blocking antibodies versus receptor inhibitors) and establish the currently omitted co-players. Increasing evidence proves that myostatin function depends on the concentration of its binding partners, e.g., activin, follistatin, and follistatin-like-3; however, they are seldom investigated in synchrony. 

## Figures and Tables

**Table 1:** Factors affecting myostatin expression in myocardium and serum.

Factor	Tissue/Cell		Serum	Reference
	mRNA	Protein		
Cyclic stretch, iv	↑	↓	-	[58]
Exercise training, rat	N	N	-	[59]
Exercise training, rat	↑	-	-	[5]
Hypokinesia, man	↑	↑	↑	[38,39]
rat	↑	↑	-	[40,41]
Angiotensin, iv	↑	↑	-	[60]
IGF-1, iv	-	↑	-	[58]
miRNA8 overexpression, mice	N	↓	-	[61]
miRNA8 deletion, mice	N	↑	-	[61]
Phenylephrine, iv	-	↑	-	[57]
Myocardial infarction, sheep	-	↑	-	[4]
rat	-	↑	-	[62]
rat	-	↑	↑	[63]
human	-	-	↓	[64]
Hypertension, rat	-	↓	-	[65]
Hypertrophy, rat	↑	↑	-	[66]
Chronic heart failure, human	-	-	↓	[67]
human	-	-	↑	[68,69,70]
human-F	-	↑	-	[71]
human-M	-	N	-	[71]

Legend: ↑—increase; ↓—decrease; N—no effect; ‘-’—not determined; iv—in vitro; F—females; M—males.
